# Multiple Membrane Interactions and Versatile Vesicle Deformations Elicited by Melittin

**DOI:** 10.3390/toxins5040637

**Published:** 2013-04-17

**Authors:** Tomoyoshi Takahashi, Fumimasa Nomura, Yasunori Yokoyama, Yohko Tanaka-Takiguchi, Michio Homma, Kingo Takiguchi

**Affiliations:** 1 Division of Biological Science, Graduate School of Science, Nagoya University, Chikusa-ku, Nagoya 464-8602, Japan; E-Mails: takahashi.lipo@gmail.com (T.T.); yohko-tk@sd6.so-net.ne.jp (Y.T.-T.); g44416a@cc.nagoya-u.ac.jp (M.H.); 2 Department of Biomedical Information, Division of Medical Devices, Institute of Biomaterials and Bioengineering, Tokyo Medical and Dental University, 2-3-10 Kanda-Surugadai, Chiyoda, Tokyo 101-0062, Japan; E-Mail: nomura.bmi@tmd.ac.jp; 3 Department of Applied Physics, Graduate School of Engineering, Nagoya University, Chikusa-ku, Nagoya 464-8603, Japan; E-Mail: yokoyama@nuap.nagoya-u.ac.jp; 4 Structural Biology Research Center, Nagoya University, Chikusa-ku, Nagoya 464-8601, Japan

**Keywords:** melittin, membrane-deformation, giant unilamellar liposome, real-time imaging, spectroscopic analysis

## Abstract

Melittin induces various reactions in membranes and has been widely studied as a model for membrane-interacting peptide; however, the mechanism whereby melittin elicits its effects remains unclear. Here, we observed melittin-induced changes in individual giant liposomes using direct real-time imaging by dark-field optical microscopy, and the mechanisms involved were correlated with results obtained using circular dichroism, cosedimentation, fluorescence quenching of tryptophan residues, and electron microscopy. Depending on the concentration of negatively charged phospholipids in the membrane and the molecular ratio between lipid and melittin, melittin induced the “increasing membrane area”, “phased shrinkage”, or “solubilization” of liposomes. In phased shrinkage, liposomes formed small particles on their surface and rapidly decreased in size. Under conditions in which the increasing membrane area, phased shrinkage, or solubilization were mainly observed, the secondary structure of melittin was primarily estimated as an α-helix, β-like, or disordered structure, respectively. When the increasing membrane area or phased shrinkage occurred, almost all melittin was bound to the membranes and reached more hydrophobic regions of the membranes than when solubilization occurred. These results indicate that the various effects of melittin result from its ability to adopt various structures and membrane-binding states depending on the conditions.

## 1. Introduction

The transformation of membranes is an essential process for various biological activities, such as cytokinesis, cell motility, endocytosis, exocytosis and maintaining the functions of organelles in living cells. Fusion and pore-formation, which accompany topological changes of membranes, are essential processes for both the infection of pathogens and the defense of hosts. The basic structure of the biological membrane is a lipid bilayer, and a number of factors, such as proteins and peptides, are involved in determining the morphology and/or stability of the lipid bilayer. Thus, it is very important to investigate the basics of interactions between lipid bilayer membranes and proteins or peptides and to understand the mechanism for regulating membrane morphology and topology. 

Melittin is an amphipathic 26-amino acid peptide toxin that is the major component of bee venom, which binds to erythrocyte membranes and causes hemolysis [1,2,3,4,5,6,7]. Although it is a small peptide, melittin exhibits a variety of effects on lipid bilayer membranes, such as the deformation of vesicles, the formation of channels or pores, fusion and disruption, or lysis [8,9,10,11,12,13,14,15]. Therefore, it has been widely studied as a model to investigate interactions between peptides and membranes [2,3,4,8,16,17,18,19,20,21,22]. The structure of melittin in solution is disordered (or a random coil) at neutral pH. When the pH, salt strength and melittin concentration of the solution are high, or when melittin binds to the membrane, melittin assumes an α-helix. Under conditions where melittin is in an α-helix structure, it forms a tetramer because electrostatic repulsion among the peptides is canceled [2,23,24,25]. This oligomer formation may be important for the formation of membrane pores by melittin in either the classical cylindrical model or in the recent toroidal model [26,27]. Although much is known about the structure of membrane-binding melittin and the mechanism of melittin-induced membrane solubilization, the mechanism whereby melittin induces a variety of changes in the membranes has not yet been elucidated, most likely because most previous studies used averaged signals obtained from bulk solution to analyze the interaction between melittin and membrane. 

In this study, we monitored the behavior of giant unilamellar liposomes during interactions with melittin using dark-field optical microscopy. Giant liposomes are cell-sized (≥1 μm) vesicles enclosed by a phospholipid bilayer that are large enough to be directly observed with optical microscopes. Giant liposomes have been used as models of biological membranes in biochemical and biophysical studies due to their similarities to actual cellular membranes in terms of both size and structure [28,29,30,31]. Optical dark-field microscopy provides real-time, high-contrast images of the intact three-dimensional morphology and dynamic behavior of individual giant liposomes in solution [32,33]. Moreover, dark-field microscopy does not require any labeling for the observation of giant liposomes, and thus, the imaging technique can be used for non-invasive long-term observations [34,35]. In addition to the knowledge obtained from the real-time imaging of changes caused in individual liposomes by melittin, for each condition studied, we characterized: (i) the predicted secondary structure of melittin using circular dichroism (CD); (ii) the membrane-binding of melittin measured using a cosedimentation assay; (iii) the degree of insertion of melittin into the lipid bilayer membrane as estimated by fluorescence quenching of the hydrophobic tryptophan residue at position 19 of the peptide, and (iv) the ultrastructure of transformed liposomes using electron microscopy (EM). Based on these results, the effect of melittin on liposomes was found to depend on the content of negatively charged phospholipids in the membrane. Depending on the conditions, melittin can take various secondary structures including an α-helix, a disordered structure (or random coil), and another structure exhibiting a β-structure-like CD spectrum. 

## 2. Results and Discussion

Giant liposomes were prepared from mixtures of phospholipids containing electrically neutral phosphatidylcholine (PC) and various molar ratios of acidic phosphatidylglycerol (PG) [36]. The lipid compositions of the prepared liposomes were: PC alone (termed PC liposomes), molar ratios between PC and PG of 9:1, 7:3, 1:1 and 3:7 (termed 10%, 30%, 50% and 70% PG liposomes, respectively), and PG alone (termed 100% PG liposomes). The PG used in this study is commonly found in the membranes of bacteria but not in those of eukaryotic cells. However, we used it as a representative of negatively charged phospholipids, e.g., phosphatidylserine and inositol phospholipids, because PG has been frequently used in experiments as a representative of acidic phospholipids, and the preparation of giant liposomes is efficiently improved if their lipid composition includes PG.

Generally, to analyze the effects of proteins or peptides on liposomal behaviors, the concentrations of protein or peptide, rather than the liposome (*i.e.*, lipid concentration), is varied. However, a number of previous studies have reported that melittin changes its activity, structure (between disordered structure and α-helix) and association state (between monomer and tetramer), depending on its concentration [23,24,25]. However, the aim of this study was to investigate the mechanism of changes in the structure and activity of melittin resulting from interactions with lipid bilayer membranes. Thus, to vary the molecular ratio between the peptide and lipid, we altered the liposome concentration but maintained a constant melittin concentration, unless otherwise noted. 

### 2.1. Melittin-Induced Liposome Transformation

To observe the alteration process from the start, giant liposomes were observed in a concentration gradient of melittin. By observing liposomes in a concentration gradient of melittin, we can observe their behavior during the transformation, and can obtain the same efficacy as if the observations were simultaneously performed at continuously changed melittin concentrations. This method enables us to find novel liposome deformations including transient responses, even if the optimal conditions for observing these are unknown and the deformations occur only under a narrow range of conditions. Finding phased shrinkage and the formation of condensed liposomes is one of the outcomes of this study. 

**Figure 1:**
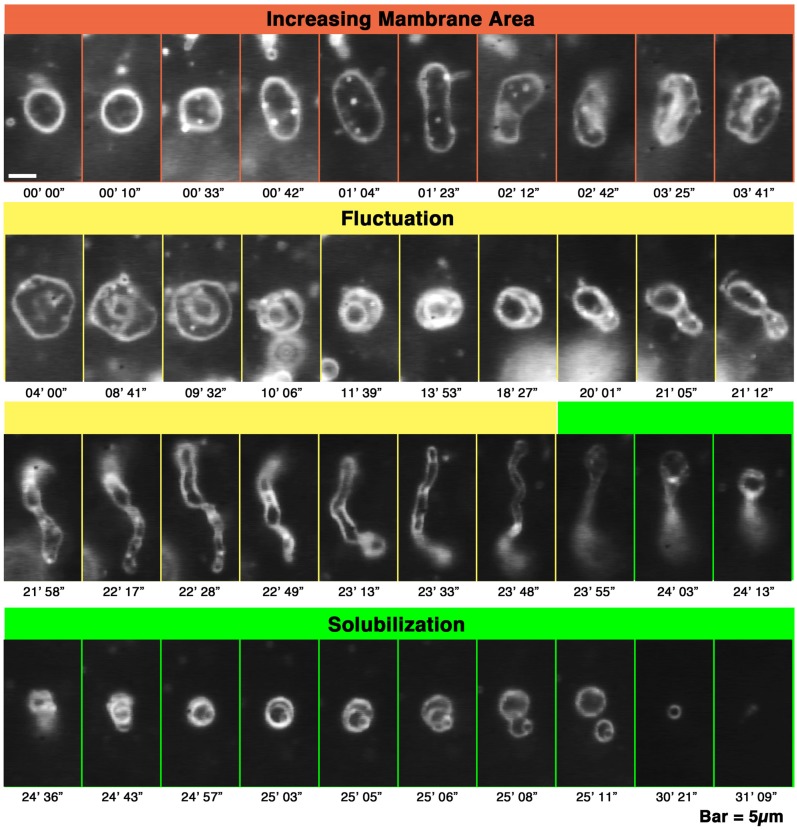
Deformation processes, increasing membrane area, fluctuation and solubilization of giant liposomes observed in a melittin concentration gradient. Time-lapse images of the deformation of PC liposomes perfused with melittin (final concentration 150 μM). The molecular ratio between melittin and the liposomes (P/L ratio), which is obtained using the ratio between the final concentrations of the peptide and lipids, is 1/4.7. The time after the start of observation is denoted in minutes and seconds under each dark-field image. The bar represents 5 μm. In figures showing the liposomal behaviors observed in a concentration gradient of melittin, the left side of each photograph is the direction in which the melittin concentration is higher.

**Figure 2:**
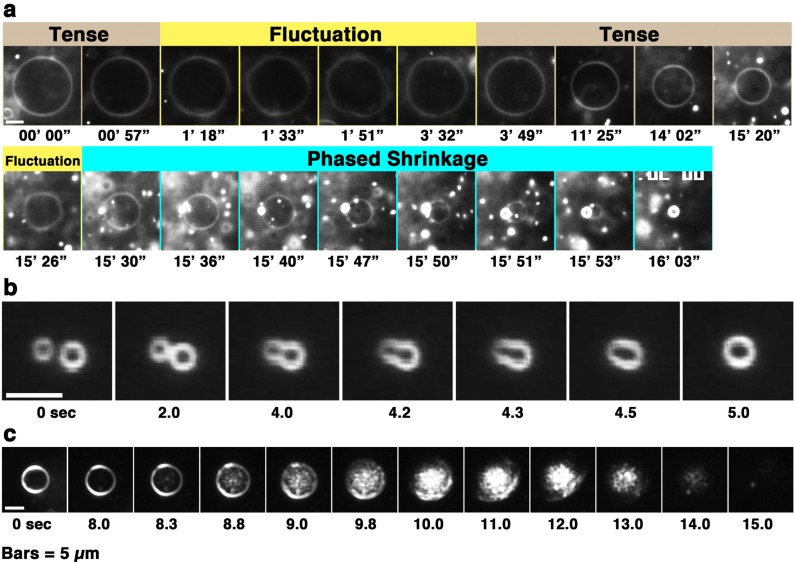
(**a**) Phased shrinkage of giant liposomes observed in a melittin concentration gradient (see also supplementary film). The condensed liposome is the product of phased shrinkage deformation; (**b**) and (**c**) represent fusions between condensed liposomes and disassembly of a condensed liposome, respectively. Time-lapse images of 50% PG liposomes perfused with melittin (final concentration: 60 μM (the P/L ratio is 1/12)). The video camera sensitivity was decreased arbitrarily according to the increase in brightness of the liposome. The time after the start of observation is denoted in minutes and seconds (**a**) or as seconds (**b** and **c**) under each dark-field image. The bars represent 5 μm. In figures showing the liposomal behaviors observed in a concentration gradient of melittin, the left side of each photograph is the direction in which the melittin concentration is higher.

First, the liposome changes induced by melittin were classified. Depending on the experimental conditions, we observed an “increasing membrane area”, “phased shrinkage” or “solubilization” of liposomes. Increasing membrane area is attributable to the intrusion of peptides into the membrane. By increasing membrane area, liposomes unequivocally increased their membrane surface area, but not their internal volume. This imbalance between surface area and volume may decrease tension, thereby resulting in vigorous fluctuations of the membrane. As a result, a spherical liposome changes into a large flabby one (Figure 1, up to approximately 24 min). The phased shrinkage of liposomes, which we consider a newly found activity of melittin, involves the formation of small bright particles on the liposome surface followed by the rapid shrinking of the liposome (Figure 2a). Usually, before phased shrinkage, liposomes alternate between the fluctuating state and the tense state. During phased shrinkage, because the number of bright particles increased, thereby resulting in an increased light intensity scattered from the liposome, the liposome finally changed into a small bright liposome, which is termed a “condensed liposome”. The bright particles and condensed liposomes are significantly different from usual membrane vesicles in brightness. Because the intensity of light scattered from liposomes and other particles depends on their density of mass [37], their appearance is consistent with the result of ultrastructural analysis showing that condensed liposomes are densely packed lipid aggregates, as will be mentioned later (Section 2.6.3). By diffusion, a condensed liposome occasionally reached a region in the specimen where the melittin concentration was much higher. In such cases, fusion between condensed liposomes (Figure 2b) or disassembly of condensed liposomes (Figure 2c) could be observed. Through solubilization, liposomes became faded and finally disappeared completely (Figure 1, times after 24 min). Sometimes, solubilization was accompanied by shrinkage and/or segmentation of the liposome. 

**Figure 3:**
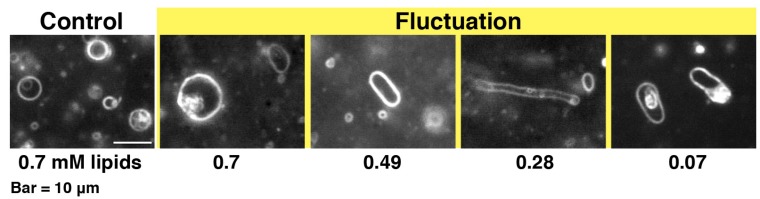
Dark-field images of 10% PG liposomes in the presence of melittin. The samples were prepared in the same way as those used for CD measurements. The final lipid concentration is denoted in mM under each dark-field image. The final melittin concentration was 60 μM (P/L = 1/12 to 1/1.2). The bar represents 10 μm.

**Figure 4:**
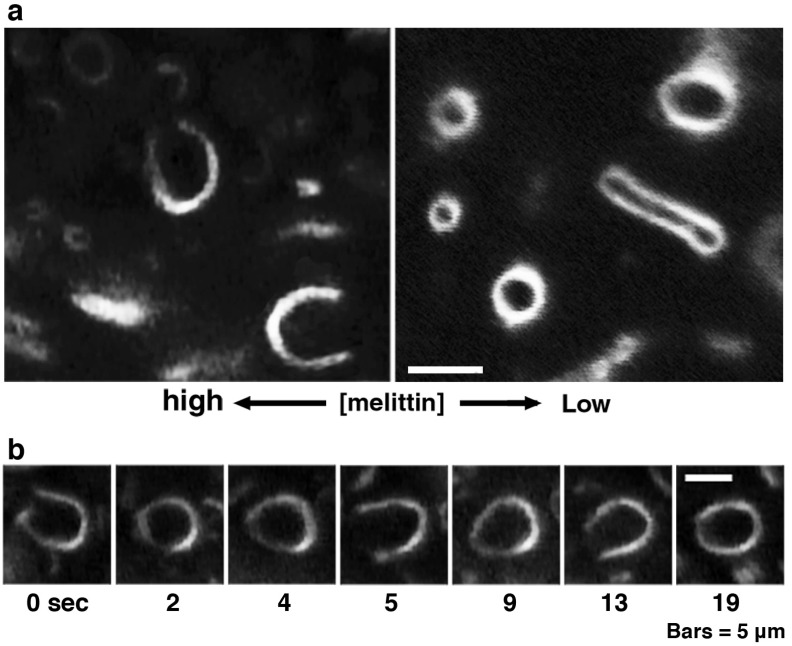
Large pore formation of giant liposomes observed in a melittin concentration gradient. PC liposomes were perfused with 1 mM melittin (P/L = 1/1). (**a**) The regions in a microscopic specimen where the melittin concentrations are low (right) and high (left) are shown, respectively; (**b**) Time-lapse images of a liposome in which a large pore has opened are shown. The time after the start of observation is denoted in seconds under each dark-field image. The cup-like shape of the liposome was unstable, and repeated opening and closing of the pore was observed. The bars represent 5 μm. In figures showing the liposomal behaviors observed in a concentration gradient of melittin, the left side of each photograph is the direction in which the melittin concentration is higher.

**Figure 5:**
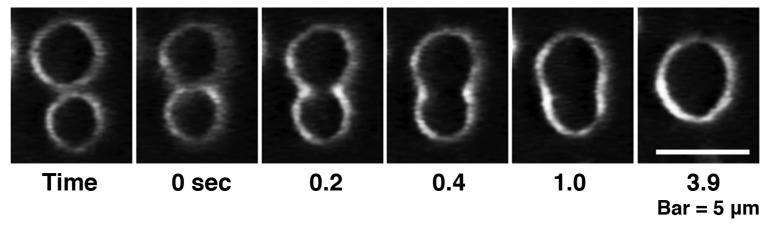
Fusion between PC liposomes observed in the presence of high concentrations of melittin. PC liposomes were perfused with 1 mM melittin (P/L = 1/1). The time after the start of fusion is denoted in seconds under each dark-field image. The bar represents 5 μm. In figures showing the liposomal behaviors observed in a concentration gradient of melittin, the left side of each photograph is the direction in which the melittin concentration is higher.

When melittin was added to PC liposomes or to 10% PG liposomes, membrane fluctuations or pearling transformations were usually observed (Figure 3), such that no drastic deformations accompanying a topological change were found. However, if higher concentrations of melittin (final 0.15–0.30 mM) were perfused, increasing membrane area progressed slowly, and, subsequently, the liposome exhibited solubilization (Figure 1). In addition, some PC liposomes opened a large pore (Figure 4) or fused with other liposomes (Figure 5) in the presence of such higher concentrations of melittin. The same formation of large pores in liposomes can be caused by proteins belonging to the band 4.1 superfamily, which have the FERM domain at their *N*-terminal. It has been reported that the FERM domain has an amphiphilic region that is similar to melittin [38,39]. These proteins are localized at the verge of the pore [40,41], suggesting that melittin also localizes at the verge of membrane-opening sites. Pores opened with melittin were very unstable, and the pore size and the entire shape of the pore-opened liposome fluctuated vigorously. Most likely, melittin molecules that are participating in pore formation lined up together only loosely at the pore verge, suggesting that the toroidal model, rather than the cylindrical model, better represents the mechanism of melittin-induced membrane pore formation because the latter model supposes tight cylindrical packing among the membrane-bound peptides [26,27]. In addition, the barrel stave pore model can also be excluded because the bilayer remains intact and the liposome structure is not perturbed in the barrel stave model [42]. Other studies, which investigated the membrane-interacting mechanism of melittin or of other pore-forming peptides, such as antimicrobials, also support the toroidal model [43,44]. The fusion between PC liposomes mentioned here is distinguishable from that between condensed liposomes because there is no preceding reaction, *i.e.*, shrinkage or increasing brightness.

**Figure 6:**
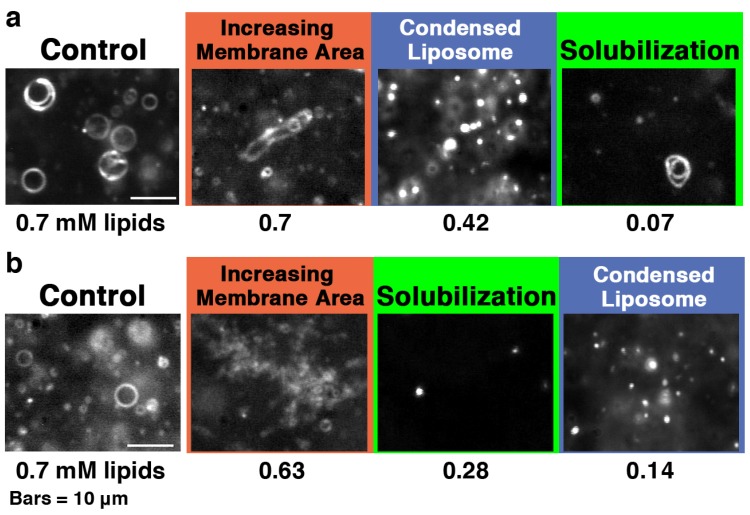
Dark-field images of (**a**) 30 and (**b**) 100% PG liposomes in the presence of melittin. The samples were prepared in the same way as those used for CD measurements. The final lipid concentration is denoted in mM under each dark-field image. The final melittin concentration was 60 μM (P/L = 1/12 to 1/1.2). The bars represent 10 μm. The video camera sensitivity was decreased arbitrarily to observe condensed liposomes.

When the concentration of PG in the liposome membrane was in the range from 30% to 100%, we could observe various liposome deformations caused by the addition of melittin. When melittin was added to 30% and 50% PG liposomes with increasing molecular ratios of peptide to lipid, the behavior mainly observed or liposome deformation changed in the order of increasing membrane area, condensed liposome formation and solubilization (Figure 6a). For 70% and 100% PG liposomes with increasing ratios of peptide to lipid, increasing membrane area, solubilization and condensed liposome formation were mainly observed, in that order (Figure 6b). In each experimental condition, the upper limit of the molecular ratio of total lipids to melittin required to observe the formation of condensed liposomes decreased with increasing PG concentration in the liposome membrane.

### 2.2. Secondary Structure of Melittin Predicted by CD Spectrometry

CD spectrometry, as well as cosedimentation, fluorescence quenching and EM observations, are not useful to study solutions involving concentration gradients. Therefore, in the following experiments, mixtures of liposomes and melittin solutions were examined at 30 min after mixing in test tubes. The solutions were observed by dark-field microscopy after the CD measurements, and we confirmed that the liposome morphologies observed were indistinguishable from those of liposomes that were deformed in a melittin concentration gradient. Additionally, solutions sampled after the fluorescence quenching experiment, aliquots of solutions prepared for the cosedimentation assay and solutions prepared before the EM observation were used to confirm liposome deformations in the same way. We note that condensed liposomes tend to aggregate on the surface of a substrate. However, no adhesion of liposomes to the surface of the cylindrical quartz cell used to measure CD was noted by direct observation using dark-field microscopy. 

**Figure 7:**
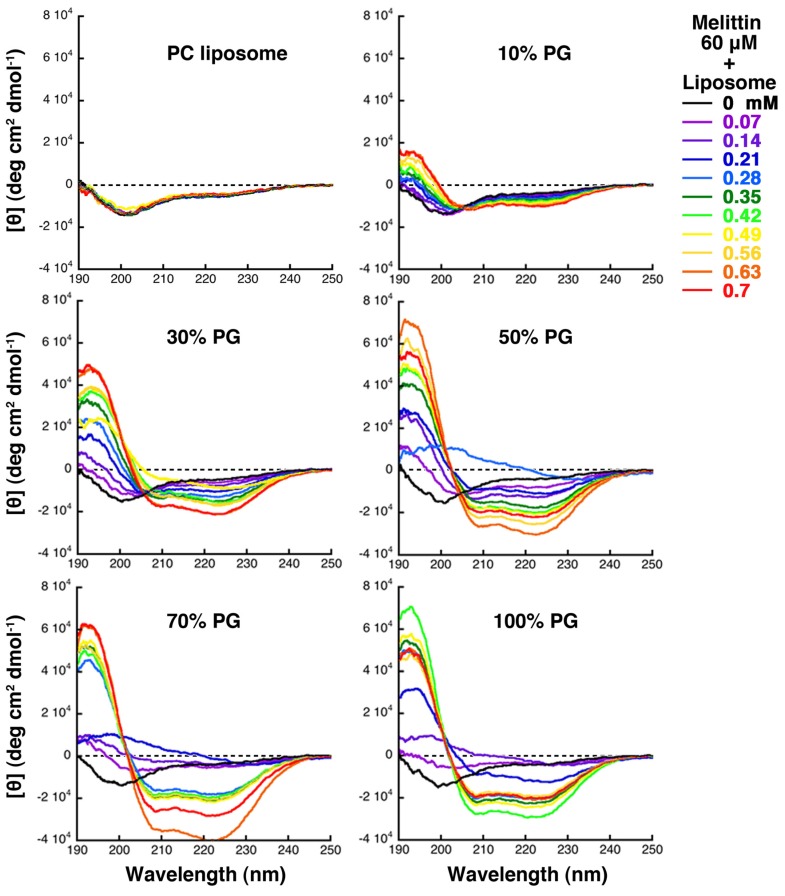
CD spectra of melittin (final concentration 60 μM) and giant liposome mixtures. The liposomes examined (PC, 10% PG, 30% PG, 50% PG, 70% PG or 100% PG liposomes) are indicated at the top of each panel. The final lipid concentration for each measurement is indicated by the color of line, as denoted on the right (P/L = 1/12 to 1/1.2).

The CD determinations showed that melittin was a mixture of peptides taking various secondary structures, such as a disordered structure and an α-helix, or a disordered structure and an another structure, which exhibited a β-structure-like CD spectrum possessing a positive band at approximately 200 nm in the difference spectrum (spectrum of melittin in the presence of each concentration of the liposomes minus that of melittin alone). However, the majority of melittin exhibited an α-helix structure, a β-structure-like CD spectrum, and a disordered structure, when assessed by CD under conditions where increasing membrane area, condensed liposome formation, and solubilization, respectively, were mainly observed (Figure 7). 

**Figure 8:**
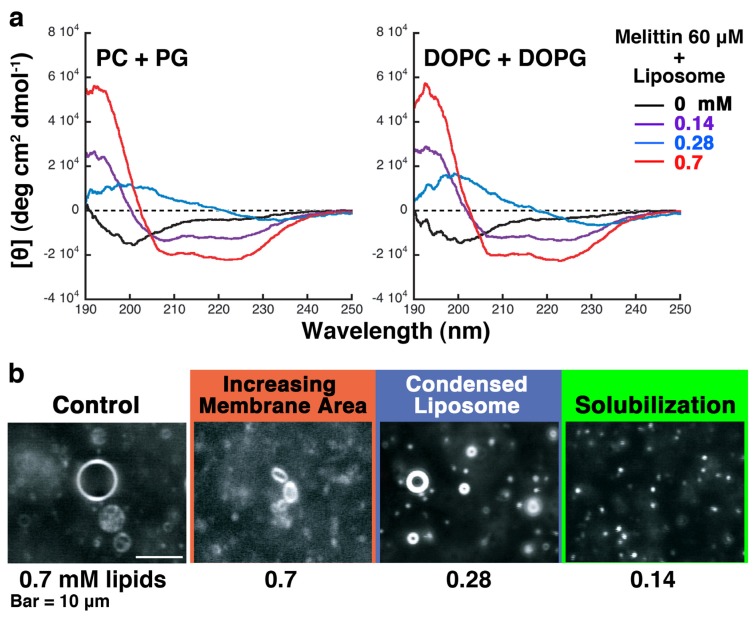
(**a**) CD spectra obtained from mixtures of melittin (final concentration 60 μM) and 50% PG liposomes (left) or liposomes prepared from DOPC and DOPG (right). The lipid concentrations are indicated by the color of the lines, as denoted on the right; (**b**) Liposomes prepared from DOPC and DOPG in the presence of melittin; the conditions used are similar to those described in Figure 6. The bar represents 10 μm. The video camera sensitivity was decreased arbitrarily to observe condensed liposomes.

When melittin was added to 10% PG liposomes, the CD spectra showed an isometric point near 205 nm, indicating a two-state transition between a disordered structure and an α-helix, and increasing helicity with increasing lipid concentration. When melittin was added to 30% PG liposomes, even though the isometric point was present, the CD spectra in the presence of 0.42, 0.49, and 0.56 mM concentrations of lipids deviated from that point, suggesting the presence of other states. When the content of PG was greater than 50%, the deviation of the spectra from the isometric point became more prominent, and the CD spectra showed that melittin forming the structure exhibiting the β-structure-like CD spectrum increased transiently. The same results were obtained even when DOPC and DOPG were used in the preparation of the liposomes rather than PC and PG, as will be discussed in the next section (Figure 8). 

The formation of condensed liposomes was observed in parallel to the emergence of the melittin fraction exhibiting the β-structure-like CD spectra. CD spectra obtained under conditions where condensed liposomes are formed indicate that melittin forms the secondary structure showing the β-structure-like CD spectrum possessing a positive band approximately 200 nm in the difference spectrum as described above. It has previously been reported that melittin forms β-structure and shows very similar CD spectral changes while interacting with heparan sulfate or heparin, which is a negatively charged polysaccharide found on the surface of cells [45]. In addition, similar CD spectral changes have been observed when amyloid β peptide interacts with PG-containing membranes [46]. Taken together, we describe this alternative structure as a β-like structure hereafter for convenience. Both α-helix and such β-like structures might be commonly seen when amphipathic peptides, such as melittin, interact with lipid bilayer membranes. We note that such a β-like structure has not been reported previously for the membrane-interacting melittin. The very high P/L ratios used here may have affected the finding of the β-like structure in this study. In any case, this finding shows that the direct real-time imaging of liposome behavior is very useful for studying interactions between membranes and peptides. 

In this study, we determined the secondary structure of melittin using CD. However, the higher order structure and dynamic behavior of the peptide were not observed. In particular, under conditions where the secondary structure of melittin is predicted to adopt β-like structure (*i.e.*, phased shrinkage), the study remains insufficient. By varying the experimental conditions more precisely or using NMR and/or calorimetry, the effect of neutralizing the charges on membrane deformation will be more defined [20,47,48,49]. Notably, large or small unilamellar vesicles should be used rather than giant liposomes when using NMR or calorimetry. Experiments using labeled melittin may also be useful if the natural activities and features of melittin are also maintained after labeling [50]. 

In general, salt is important for maintaining the structure and activity of proteins and peptides. Therefore, studies performed under conditions that are nearer to physiological salt concentrations are essential. In addition, lipid membrane morphology could be affected by environmental factors such as temperature, osmolarity and pH as well as salt strength [19,21,51,52]; thus, a considerably wider range of conditions should be examined to understand melittin activities more fully. 

### 2.3. The Effect of Substituting Phospholipids Obtained from Natural Extracts by Synthetic Phospholipids

In this study, we used natural phospholipids extracted from living cells or egg yolk to reproduce an environment similar to the *in vivo* situation. The fatty acid tails of natural phospholipids have a variety of chain lengths and saturations. However, previous studies investigating the mechanism of melittin-induced membrane deformation have usually used synthetic phospholipids that have uniform fatty acid tails. Therefore, we investigated the effects of heterogeneity in the fatty acid tails on melittin-induced liposome deformations (Figure 8).

Because all deformations can be induced using 50% PG liposomes by varying the lipid concentration, we prepared giant unilamellar liposomes made from DOPC and DOPG (1:1, mol/mol) to confirm the results. When DOPC:DOPG liposomes were used rather than 50% PG liposomes, almost the same results were obtained in real-time observations using dark-field microscopy and in CD spectra, suggesting that differences in the fatty acid tails have little effect on the interaction between melittin and liposomes. 

### 2.4. Membrane Affinity of Melittin

**Figure 9:**
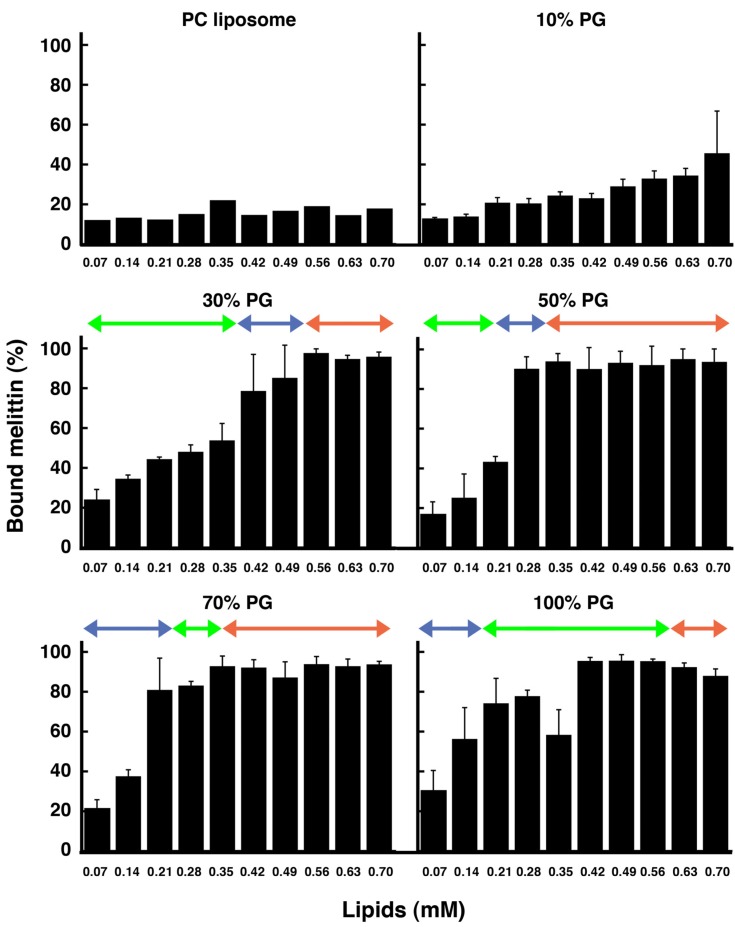
Fraction (%) of liposome-bound melittin. The final melittin concentration was 60 μM. The liposomes examined (PC, 10% PG, 30% PG, 50% PG, 70% PG or 100% PG liposomes) are indicated at the top of each panel. The experimental conditions were the same as those used for the CD measurements. Green, blue and red arrows in each panel show the ranges of concentration of total lipids where solubilization, phased shrinkage and increasing membrane area were mainly observed, respectively. Error bars indicate standard deviations.

**Figure 10:**
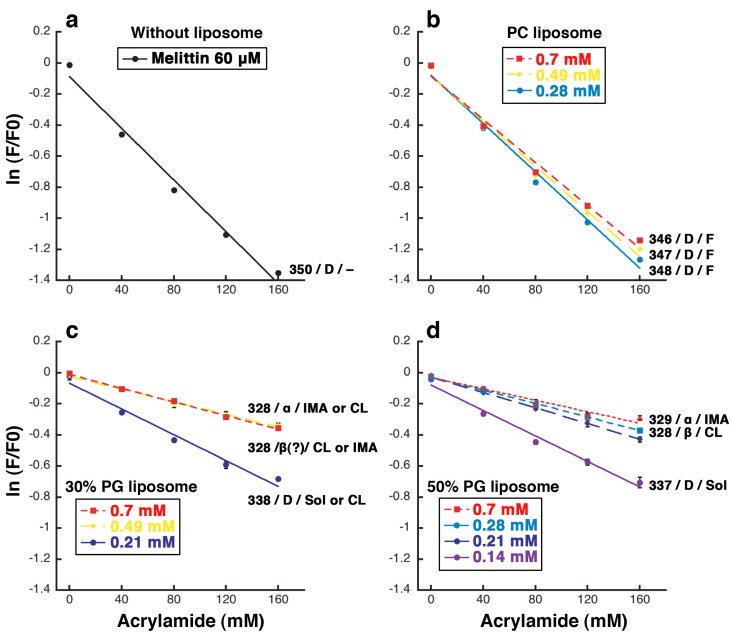
Fluorescence quenching of tryptophan residue 19 of melittin by acrylamide. The results obtained with melittin alone (**a**) or mixed with PC (**b**), 30% PG (**c**), or 50% PG liposomes (**d**) are shown. The experimental conditions were the same as those used for the CD measurements. The final melittin concentration was 60 μM. The final lipid concentration is indicated by the color of line, as denoted in the box in each panel. The wavelength (nm) of the emission maximum, the estimated secondary structure of the majority of melittin and the typically observed liposome deformation are indicated at the side of each line. A disordered structure, an α-helix, and a β-like structure are denoted as “D”, “α”, and “β”, respectively. The fluctuation of liposome, increasing membrane area, condensed liposome formation, and solubilization are denoted as “F”, “IMA”, “CL”, and “Sol”, respectively.

In each condition, the amount of membrane-bound melittin was determined by cosedimentation with liposomes (Figure 9). For PC liposomes, even though melittin bound to the membrane as reported previously [44,49], the membrane-bound fraction was approximately only 10%–20%. For 10% PG liposomes, the membrane-bound fraction slightly increased; however, the amount remained at a lower level than for the 30%, 50%, 70%, and 100% PG liposomes. Conversely, melittin bound substantially to 30%, 50%, 70%, and 100% PG liposomes (approximately 20% of melittin bound to the liposomes even when the concentration of lipids was low, and almost 100% of melittin bound to the liposomes when the concentration of lipids was high). The 30%, 50%, 70%, and 100% PG liposomes could be transformed to condensed liposomes. These membrane-bound melittin fractions, which were determined by the cosedimentation assay, are consistent with the results indicated by CD (Figure 7) and the fluorescence quenching of the tryptophan residue (Figure 10, next section). Our results indicate that the affinity of melittin to membranes containing only neutral lipids is very weak and that negatively charged lipids can significantly increase the membrane binding of melittin, as reported previously [53]. 

For the 30% and 50% PG liposomes, when the lipid concentration was lower than that required to induce phased shrinkage (*i.e.*, the transformation of liposomes to condensed liposomes), melittin was abundant in the supernatant, indicating that membrane binding of the peptide was saturated under these conditions. Under conditions where increasing membrane area or condensed liposome formation was observed, almost all melittin was bound to liposomes. Therefore, the secondary structure expected by CD under these conditions should be that of the membrane-bound melittin. 

For the 70% and 100% PG liposomes, condensed liposome formation could be observed at lower lipid concentrations than for solubilization. Under these conditions, the majority of the peptide was found in the supernatant, although condensed liposomes were formed. This discrepancy between the results of the cosedimentation assay and those of the other methods (the results of real-time observations and CD as described above and the results of the fluorescence-quenching assay as described later) may be because the liposome membranes were collapsed by that deformation, broken into fragments and, thus, no longer precipitated by centrifugation. 

### 2.5. Fluorescence Quenching of the Tryptophan 19 Residue

Melittin has a tryptophan residue at position 19. If the indole ring of that residue exists in a hydrophilic environment, *i.e.*, if melittin does not penetrate the lipid bilayer membrane, then the fluorescence from the residue would be quenched by acrylamide in an aqueous solution. Conversely, if melittin penetrates the membrane, then the fluorescence would remain and be detectable because acrylamide cannot access the indole ring in a hydrophobic environment. Therefore, based on the fluorescence intensity of the tryptophan residue, we can estimate the position of melittin in the membrane under each condition [54]. It should be noted that we have confirmed that acrylamide does not cause any changes in the morphology or topology of giant liposomes (data not shown).

When melittin is alone in solution, the fluorescence intensity from the tryptophan residue at position 19 was exponentially decreased depending on the concentration of acrylamide. The efficiency of quenching was 8.3 M^−1^ (Figure 10a), and the emission maximum was observed at approximately 350 nm. In the presence of PC liposomes, the quenching profile was similar to that of melittin alone, whereas the efficiency of quenching was slightly decreased to 6.9 M^−1^ at 0.70 mM PC liposomes (the efficiency is 17% lower than for melittin alone, Figure 10a,b), and the emission maximum was slightly changed. When the final lipid concentrations were 0.28, 0.49, and 0.70 mM, the emission maxima were approximately 348, 347, and 346 nm, respectively. Because acrylamide quenched the fluorescence intensity even in the presence of PC liposomes, the tryptophan residue was assumed to be in a hydrophilic environment, such as in solution or on the surface of the membrane. To reduce the acrylamide-induced fluorescence quenching, it appears that much higher concentrations of PC liposomes would be required. The cosedimentation assay indicated that the affinity between melittin and PC liposomes is weak and showed that approximately only 10%–20% of melittin is bound to the membrane (Figure 9). These results indicate that most melittin does not bind to membranes prepared from neutral PC alone. However, from another point of view, the results indicate that a small fraction of melittin binds to and most likely penetrates into the membrane because the non-quenching fraction in the fluorescence-quenching assay and the membrane-bound fraction in the cosedimentation assay were very similar. This binding and penetration of melittin may be a reason for the fluctuation of PC liposomes in the presence of melittin (Figure 1). In previous studies, the interaction between melittin and membranes made from PC only was reported [2,26,27,55]. In contrast, our study shows only weak binding between melittin and PC liposomes, and a high concentration of melittin is required to induce the solubilization of PC liposomes. It has been reported that melittin has a tendency to form aggregates on membranes [26,27]. Even if the membrane affinity of melittin is weak, upon binding to the membrane, melittin may develop the ability to form an aggregate on the membrane. As the amount of membrane-bound melittin exceeds a threshold, melittin immediately would form an aggregate involving the surrounding lipids and dissociate from the membrane, a process that results in the solubilization of the liposome. 

When melittin was added to 30% PG liposomes (Figure 10c), different results were obtained depending on the lipid concentration. When the final lipid concentration was 0.21 mM (the condition where solubilization or the formation of condensed liposomes can be observed), the fluorescence was obviously quenched and the emission maximum shifted to 338 nm. When the final lipid concentrations were 0.49 or 0.70 mM, conditions where the formation of condensed liposomes or increasing membrane area can be observed, only a little quenching was observed, and the emission maximum was shifted to 328 nm. When the final lipid concentration was 0.35 mM, the reproducibility of the measurements was very low, suggesting that this condition (a final lipid concentration of 0.35 mM) represents a transition between the above two conditions (lipid concentration ranges: ≤ 0.21 and ≥ 0.49 mM).

When melittin was added to 50% PG liposomes (Figure 10d), the results obtained were dependent on the lipid concentration, similar to when 30% PG liposomes were used. When the final lipid concentration was 0.14 mM (the condition where solubilization can be observed) the fluorescence was obviously quenched. In contrast, when the final lipid concentrations were 0.28 or 0.70 mM, conditions where the formation of condensed liposomes and increasing membrane area can be observed, respectively, only a little quenching was observed. When the final lipid concentrations were 0.14, 0.28, or 0.70 mM, the emission maxima were shifted to 337, 328, and 329 nm, respectively. In the presence of 50% PG liposomes, the efficiency of quenching was significantly decreased to 1.9 M^−1^ at 0.70 mM liposomes (the efficiency is approximately 80% lower than for melittin alone, Figure 10a,d). The results of the cosedimentation assay indicated that the affinity between melittin and PG liposomes is strong, as described above, and showed that approximately 90% of melittin bound to the membrane at 0.28 mM liposomes (Figure 9). Therefore, it appears that approximately 80% of melittin is incorporated into the membrane, 10% is bound to the surface, and the remaining 10% remains in solution. We note that when melittin was added to 70% or 100% PG liposomes, the reproducibility of the measurements was very low at any lipid concentration (data not shown), suggesting that a complex interaction took place between melittin and these liposomes. 

Taken together, when increasing membrane area or phased shrinkage occurred, melittin reached more hydrophobic regions of the lipid bilayer than when solubilization occurred. In view of the facts that increasing membrane area causes a large increase in membrane area and melittin opens large pores in membranes, we consider that melittin inserts not only into the outer leaflet but also translocates to the inner leaflet of the lipid bilayer. The degree of penetration depended on the concentration of negatively charged PG, but not on that of total phospholipids. 

### 2.6. Mechanism of the Deformation of Membranes Containing Negatively Charged Lipids by Melittin

#### 2.6.1. Binding of Melittin to Membranes Containing PG

Melittin caused a variety of morphological changes in liposomes containing PG in their lipid composition. Negatively charged phospholipids, such as PG, enhance the membrane binding of melittin. Moreover, negatively charged phospholipids, e.g*.*, phosphatidylserine or inositol phospholipids, as well as PG, are always contained in biological membranes and play many important roles in living cells. Thus, studies that examine the effects of PG are very important to understanding the effects of melittin on lipid membranes [49,50,53,56]. 

At the lower ratio of melittin to total lipids (the sum of PC and PG), increasing membrane area most likely occurs due to the penetration of melittin molecules into the lipid bilayer membrane as the result of electrostatic interactions because melittin penetration should increase the number of molecules constituting the membrane. That is, the membrane surface area of liposomes increases according to the degree of melittin penetration. If the inner volume remains constant, then the imbalance caused by increasing the surface area only should induce transformations of the liposomes such as tubulation or pearling. Furthermore, when the more melittin penetrates the hydrophobic region of the lipid bilayer membrane, hydrophobic interactions between the fatty acid tails of the phospholipids should be disturbed, and such a disruption would destabilize the membrane, e.g., by pore formation. 

At the higher ratio of melittin to total lipids, solubilization may be caused by a surfactant-like action of the amphiphilic melittin. Previously, it has been reported that high concentrations of melittin cause micellization of the lipid bilayer and that the secondary structure of melittin is an α-helix under this condition [1,55]. Contrarily, a disordered secondary structure of melittin is predicted under conditions in which solubilization occurred in our study (Figure 7). This discrepancy may be because very large amounts of melittin are associated with membrane surfaces that contain negatively charged lipids, and melittin was not allowed to reach a sufficient surface area to bind to the membrane by adopting an α-helix; thus, the structure should remain disordered. Alternatively, the result may be simply due to excess membrane-free melittin, *i.e.*, the signal of the CD spectrum from membrane-free melittin obscures that from membrane-bound melittin. 

When the ratio of melittin to total lipids is between the above two conditions, condensed liposomes are formed. Condensed liposomes tend to easily fuse with each other (Figure 2). 

Incidentally, because it has been reported that other pore-forming toxins show surfactant-like action and fusogenic activity similar to that of melittin [57], these features would be closely related to pore-formation. 

In all cases, the β-like structure is more frequently predicted as the secondary structure of melittin as the proportion of negatively charged PG in the liposome membrane increases (Figure 7). As discussed in the following section, PG may stabilize the β-like structure of melittin and support the formation of dense aggregations comprising β-like structured peptides and lipids by decreasing the repulsion among positively charged melittin molecules, given its negative charge. The formation of such aggregations consisting of melittin and lipids may be responsible for the phased shrinkage of liposomes, *i.e.*, the formation of condensed liposomes. 

#### 2.6.2. Effect of Offsetting the Charges

When the concentration of melittin added was constant, the amount of PG required for inducing phased shrinkage, *i.e.*, condensed liposome formation, also became almost constant because the PG content in a liposome membrane required to induce the deformation of the liposome was inversely proportional to the total lipid concentration required to induce the deformation (Section 2.1). The amount of PG was a dominant factor that determined the secondary structure of the peptide bound to the membrane (Section 2.2), the affinity of melittin to the membrane (Section 2.4), the depth of penetration of the peptide into the membrane (Section 2.5), as well as the deformation induced in the membrane by the peptide. This suggests that offsetting the positive charge of melittin by the negative charge of an acidic phospholipid would play an important role in the mechanism of membrane deformation. Most likely, the membrane-deforming activity of melittin is altered from solubilization to condensed liposome formation and to increasing membrane area in a phased manner with the increased degree of charge offset. This assumption is supported by the hypothesis that α-helix formation of the peptide is hindered by charge repulsion among basic residues that are localized at the *C*-terminus. 

Our results suggest an overview for the mechanism of lipid bilayer membrane deformation induced by melittin. Melittin can interact with both neutral phospholipids and negatively charged acidic phospholipids. When a membrane contains only neutral phospholipids, melittin interacts with the membrane only weakly and cannot adopt a stable structure. When a membrane contains acidic phospholipids, melittin interacts with the membrane tightly through strong electrostatic interactions and can form an α-helix or a β-like structure by offsetting the charges. Melittin that forms an α-helix structure may strongly exhibit an amphiphilic feature, resulting in a strengthening of its interaction with the membrane. Such an interaction might be involved in increasing membrane area, large pore formation, or the solubilization of the condensed liposome shown in this study, and physiologically might be involved in the strong hemolytic activity of melittin. Melittin that forms a β-like structure may develop the ability to aggregate. The assembled form of melittin will be a component of the dense aggregation that forms condensed liposomes. 

**Figure 11:**
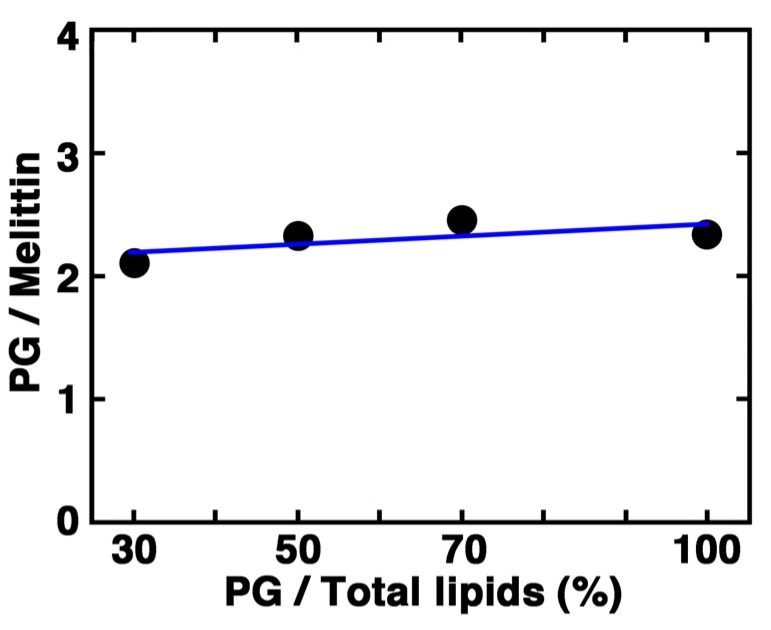
A plot of the optimum molecular ratio of PG to melittin required to observe the formation of condensed liposomes against the content (%) of PG in the liposome membrane.

The optimum molecular ratio of PG lipid to melittin required to observe the formation of condensed liposomes was almost constant and independent of the lipid composition of the liposome membrane (Figure 11). The same relationship between PG and melittin could also be obtained for affinity and the penetration depth of the membrane binding of melittin. The fact that the ratio of PG lipid to melittin, rather than that of total lipids to melittin, determines the nature of the interaction that occurs between the liposome and melittin and which deformation is induced (Figure 6, Figure 7, Figure 8, Figure 9, Figure 10), further suggests the importance of offsetting the charges [58]. Melittin contains five basic amino acid residues, and its *C*-terminus is amidated. These results suggest that approximately two acidic lipids participate in the offset. 

The relationship between the molar ratio of lipid and melittin and the liposomal deformation induced (*i.e.*, whether solubilization took place or condensed liposomes were formed) was different between the cases of 30% and 50% PG liposomes and the cases of 70% and 100% PG liposomes (Section 2.1 and Section 2.4; see Figure 6, for example). When liposome contains acidic phospholipids in excess of the concentration required to offset the positive charge of melittin, the excess negative charges of the excess acidic phospholipids may complicate the reactions of liposomes in the presence of melittin by generating a repulsive force among the molecules. 

#### 2.6.3. Ultrastructural Analysis of Condensed Liposomes Induced by Melittin

**Figure 12:**
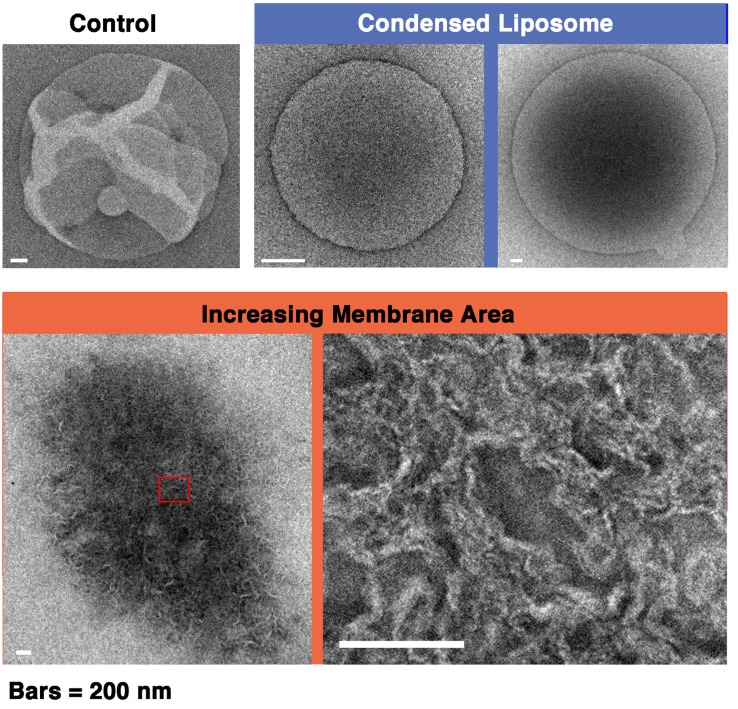
EM images of a liposome in the absence of melittin (control), condensed liposomes (two representatives shown here), and a liposome that has undergone increasing membrane area are shown. The bottom right image is an enlarged image of the boxed area on the bottom left. The bars represent 200 nm. To obtain condensed liposomes and liposomes that have undergone increasing membrane area, 50% PG liposomes (final concentrations 0.28 and 0.70 mM, respectively) were mixed with melittin (final concentration 60 μM (P/L ratios: 1/4.7 and 1/12, respectively)).

We investigated the membrane ultrastructure formed when melittin interacts with liposomes using EM (Figure 12). Under conditions where increasing membrane area takes place, lipid bilayer structures could be observed, forming dense multilayered structures. Conversely, under conditions where phased shrinkage takes place, no layered structures could be found; rather, only uniformly negatively stained droplet-like spheres were observed. These results suggest that the condensed liposome comprises a densely packed mixture of lipids and peptides rather than a folded lipid bilayer membrane, and this may be one reason why the fusion between condensed liposomes can occur easily. It should be noted that the EM observation regarding condensed liposomes may be relevant to the cryo-TEM result of a previous study that demonstrated that melittin causes the disassembly of liposomes prepared from PC and PG and the formation of vesicles without clear structures [50]. 

**Figure 13:**
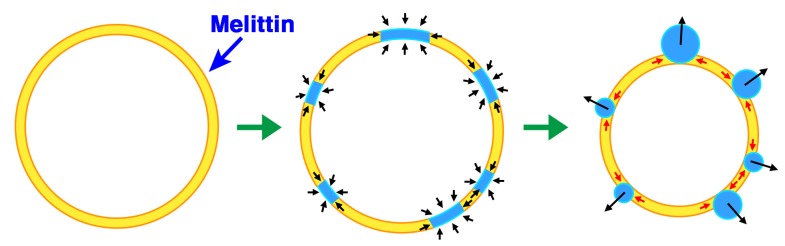
Model for phased shrinkage. The negatively charged phospholipid melittin binds to the membrane (**left**). The membrane-bound peptides form densely packed aggregates with the phospholipids, resulting in the droplet-like regions. The regions exhibit high brightness (**center**). The droplet-like regions are excluded from the surrounding lipid bilayer region by the membrane line tension. Concurrently, the droplet-like regions become spherical to reduce their surface area (**right**). As the result of repeating these processes, the liposome decreases in size to a small bright particle.

**Figure 14:**
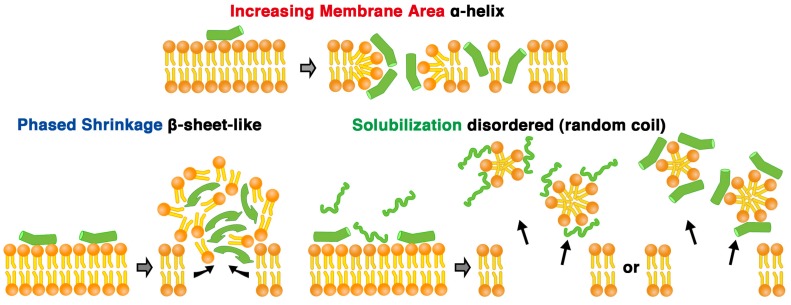
Schematic illustration of melittin-induced membrane deformation and the structure of melittin. The secondary structure of the majority of melittin was estimated as an α-helix, a β-like structure or a disordered structure under conditions where increasing membrane area, phased shrinkage or solubilization are mainly observed, respectively. At first, melittin binds to the surface of the membrane, possibly in parallel. Subsequently, with increasing concentrations of the peptide, melittin sequentially induces disassembly of the bilayer structure and solubilization of the liposome (bottom right), or penetrates into the membrane. The membrane-penetrating peptides increase membrane area (top). The membrane-binding or -penetrating peptides most likely interact with each other [44,56] and form pores (top) or form aggregates with phospholipids, causing the formation of condensed liposomes (bottom left). In the case of solubilization, two considerable cases (the secondary structure of melittin is a disordered or an α-helix) are illustrated.

Taken together, the results indicate that phased shrinkage can be visualized as follows: As melittin is bound to the lipid bilayer membrane, a droplet-like region appears in the membrane, and the structure of the droplet-like region appears to indicate that phospholipid molecules do not form any ordered structures such as bilayers. Because the droplet-like region has no obvious hydrophobic part, this region will be excluded from the surrounding region that retains the normal lipid bilayer structure. To be more stable, *i.e.*, to reduce the surface area in contact between two regions, the droplet-like region become spherical. At the same time, the region that maintains the bilayer structure contracts while eliminating the droplet-like regions by line tension (Figure 13, see also Figure 14). 

#### 2.6.4. Comparison with Condensed Liposomes That Are Induced by Fusionegic Peptides

Influenza hemagglutinin is a membrane-fusion-inducing protein that is essential for the influenza virus infection. We previously have found that a pair of positively and negatively charged synthetic peptides (HA peptides) derived from the *N*-terminus of the influenza hemagglutinin HA2 subunit induces phased shrinkage-like deformation [33]. As a result, giant liposomes were altered to condensed liposome-like products. However, EM observation showed that the condensed liposome-like product comprised densely folded lipid bilayers but not lipid aggregates. Although the transformation processes induced by HA peptides are similar to those induced by melittin, their mechanisms are most likely different from each other. In the case of melittin, the positive charge of the peptide molecule is neutralized with the counter negative charge of lipid molecules in the membrane, which binds the peptides through electrostatic interactions. However, for HA peptides, the positively and negatively charged peptides neutralize each other, and lipids in the membrane are not directly involved in the charge offset [59]. 

Unlike melittin, HA peptides can only induce the phased shrinkage-like deformation of liposomes and subsequent fusion between the condensed liposome-like products [33]. Therefore, the formation of condensed liposome-like products might be the membrane-deformation commonly seen when amphipathic peptides interact with liposomes. Conversely, these results indicate that amphiphilic nature and charge are insufficient to make a peptide exert the various membrane-deforming activities observed. The *N*-terminus of the influenza hemagglutinin HA2 subunit adopts an α-helix in the soluble state [60,61,62], and the α-helical structure breaks in the membrane-bound state [63,64,65,66,67,68,69]. However, melittin can take various secondary structures; an α-helix, a β-like structure or a disordered structure. This higher degree of freedom found in the structure of membrane-interacting melittin is important for inducing various membrane deformations (Figure 14). Our study reveals that peptides and lipid membranes can interact in a variety of ways. The membrane interactions of other many peptides, toxins, and antimicrobials are most likely sustained by a variety of such mechanisms [43,57]. 

## 3. Experimental Section

### 3.1. Lipids and Melittin

Natural phospholipids extracted from egg yolk, PC, PG, synthetic phospholipids, DOPC, DOPG and melittin (a natural extract greater than 85% pure and a synthetic peptide greater than 97% pure) were purchased from Sigma (St. Louis, MO, USA). The melittin concentration was calculated using the molar absorption coefficient at 280 nm (5550 M^−1^ cm^−1^). Because the same liposome deformations were observed with either type of melittin, we used the natural extract in this study, unless otherwise noted. 

### 3.2. Preparation and Observation of Liposomes

Liposomes were prepared as described previously [32,37,70]. Lipid films prepared in test tubes from 500 nmol of phospholipids were added to 500 μL buffer A (10 mM HEPES-NaOH, pH 7.0) (denoted as 1 mM liposome or 1 mM lipids) and incubated at 50 °C for 2 h to form liposomes. To prepare liposomes from synthetic phospholipids, PC and PG were substituted by DOPC and DOPG, respectively. 

Direct real-time imaging of liposomes in a concentration gradient of melittin using dark-field microscopy was performed as described previously [37]. To prepare a concentration gradient of melittin suitable for examination under a microscope, we used a mixing chamber comprising a glass slide and a cover slip, which were firmly fixed together with spacers. This chamber has three open channels. To prepare a concentration gradient of the peptide in a liposome solution, a drop of the melittin solution was placed in one open channel of the chamber, and the liposome solution was placed in another channel. These two solutions seeped into the chamber, and air exited through the third channel. The solutions gently attached and mixed with each other, thereby forming a concentration gradient through diffusion. We manually tracked the flowing liposomes in focus, recorded the images, and analyzed them with Photoshop (Adobe Systems) or ImageJ software [32,33,34,37]. 

For ultrastructural analysis of condensed liposomes and liposomes that have undergone increasing membrane area, 0.40 and 1.0 mM 50% PG liposomes were mixed with 0.20 mM melittin (volume ratio, 7:3), respectively. Then, each sample (after liposomal morphology was pre-observed by dark-field microscopy) was adsorbed onto formvar-coated copper grids, negatively stained with 2% uranyl acetate and observed using a transmission electron microscope, JEM-1200 EX II (JEOL Ltd., Tokyo, Japan) [34,35,37]. 

### 3.3. Circular Dichroism

The prepared liposomes were mixed with 0.20 mM melittin (volume ratio, 7:3), and samples were then incubated for 30 min after brief mixing using a vortex mixer. CD spectra were collected using a CD spectrometer (J-700, JASCO, Hachioji, Tokyo, Japan) with a cylindrical cell of 0.20 mm optical path length at room temperature. Each CD spectrum was recorded at a scan rate of 20 nm min^−1^, a 1.0-nm band-path, and a response time of 8.0 s; 4 scans were averaged. The mean residue molar ellipticity was determined from the obtained data. An aliquot of each sample and/or each sample after the CD measurements were observed by dark-field microscopy to confirm that morphological changes had occurred. 

### 3.4. Cosedimentation Assay

The cosedimentation of melittin with liposomes was performed as described previously [41]. Samples (total volumes of 100 μL) were prepared as described in Section 3.3 and centrifuged at 160,000 × *g* (TLA-100.1 rotor, 65,000 rpm) for 30 min at 25 °C. The separated samples were resolved using SDS-PAGE by applying the samples to preformed gels (NuPAGE (R) 4%–12% Bis-Tris Gel, Invitrogen, Carlsbad, CF, USA). Melittin bands were silver-stained (Silver Stain II Kit Wako, Wako Pure Chemical, Osaka, Japan), and their intensity was measured using ImageJ software. 

### 3.5. Fluorescence-Quenching Experiments of the Tryptophan Residue of Melittin

After the concentration of liposomes was arbitrarily adjusted with buffer A, each liposome solution was mixed with 0.20 mM melittin (volume ratio, 7:3) and then incubated for 30 min as described above. The fluorescence intensity from the tryptophan residue at position 19 of melittin was measured using a spectrofluorometer (FP-6500, JASCO) at 25 °C. Each fluorescence spectrum was recorded using a scan rate of 50 nm min^−1^, 10-nm band-paths for excitation and fluorescence, an excitation wavelength of 280 nm, a fluorescence wavelength of 300–450 nm, a response time of 8.0 s, 1.0 nm of data acquisition, and high sensitivity.

The fluorescence spectrum (F0) of a mixture of liposomes and melittin was measured; subsequently, acrylamide was added (final concentration: 0, 40, 80, 120 or 160 mM), and the spectrum was recorded again. The concentration correction value considering the addition of acrylamide (F) was calculated using the accumulated value of the fluorescence intensity in the range of 300–400 nm, after subtracting the background. From these obtained values, ln (F/F0) was calculated. 

## 4. Conclusions

Kinetic analysis by EM observations and imaging using fluorescent probes are powerful and effective approaches to investigate the already well-known transformation events of membranes, such as fusion or channel formation. However, if an unknown deformation process is involved in the morphogenesis and dynamics of membranes, these methods will not reveal certain mechanisms due to their static and indirect detection principles. In this study, the real-time imaging of giant liposomes using dark-field optical microscopy successfully revealed that melittin induces a variety of deformations, increasing membrane area, phased shrinkage, and solubilization of liposomes, as well as pore formation and fusion, were observed depending on the conditions. The concentration of acidic phospholipids included in the membrane and the molecular ratio between the liposome and melittin are important for determining the structure and activity of melittin. The secondary structure of melittin was estimated as an α-helix, a β-like structure, or a disordered structure for conditions where increasing membrane area, phased shrinkage, or solubilization is observed, respectively. When increasing membrane area or phased shrinkage occurred, almost all melittin was bound to the membranes, and melittin reached more hydrophobic regions of the membrane compared to that seen with solubilization. These results indicate that melittin exhibits a variety of effects because this peptide can take various structures and membrane-binding states depending on the conditions. Offsetting the positive charge of melittin by the negative charge of an acidic phospholipid can determine the nature of the interaction that occurs between the liposome and melittin, and would play an important role in the mechanism of membrane deformation. 

## Supplementary Files

Supplementary File 1 Supplementary Materials (ZIP, 2599 KB)
