# Supplementary material for: Multiple Membrane Interactions and Versatile Vesicle Deformations Elicited by Melittin
Source: Toxins (Basel). 2013 Apr 17;5(4):637–64. doi: 10.3390/toxins5040637 (PMC3705284; doi:10.3390/toxins5040637)
Supplement: Supplementary File 1 — Supplementary Materials (ZIP, 2599 KB) [file toxins-05-00637-s001.zip › CaptionOfSupplementaryMovie.pdf]

The Caption of Supplementary Movie

Phased Shrinkage of a giant liposome observed in the concentration gradient of melittin by dark-field microscopy. 50% PG liposomes (final concentration 0.7 mM) were perfused with melittin (final concentration 60 μM). Movie is played by real-time speed. Approximately 20 seconds after the start of Movie, the video camera sensitivity was decreased arbitrarily according to the increase in brightness of the liposome.

It should be noted that, sometimes Condensed Liposomes, which had been already formed, are seemed as if absorb to the surface of a liposome that is progressing Phased Shrinkage. Many of them, however, only just happen to be close or to collision to the liposome. In fact, these Condensed Liposomes were usually repelled after the collision. Almost of the bright spots emerged newly on the surface of liposome in progress on Phased Shrinkage. Frequent adhesion and fusion between Condensed Liposomes were observed in the area where the concentration of melittin was higher.
